# 

**Published:** 1891-12

**Authors:** 


					﻿VOL XI.
DECEMBER, 1891.
NO. 12
IPHE
HOMEOPATHIC PHYSICIAN,
A Monthly Journal of Homoeopathic Materia Medica
and Clinical Medicine.
“ He is a freeman whom truth makes free, and all are slaves beside.”
” Seek the Truth: come whence it may, cost what it will.”
EDITED AND PUBLISHED BY
WALTER Ni. JAMES, M. D.
PHILADELPHIA :
No. 1125 SPRUCK STREET.
L O N D O N '•
ALFRED HEATH & CO., Sole Agents fob Gbeat Britain,
114 Ebury Street, Eaton Square.
9&~'Xearly Subscription, $2.50. invariably in advance. Single numbers, 25 ets.
Entered at the Philadelphia Poet-Office aa Second-Claae Mail Matter.
				

